# Anti-human T lymphocyte porcine immunoglobulin attenuates experimental autoimmune encephalomyelitis by inhibiting T cell activation

**DOI:** 10.1038/s41598-026-55508-7

**Published:** 2026-05-30

**Authors:** Junchen Liu, Xilin Nie, Fan Yang, Peng Geng, Jingwen Jin, Xuan Liu, Lingchen Kong, Yipeng Zhao, Wenqian Mao, Qianwen Ma, Yinghao Song, Jiaxuan Wu, Ruizhu Wang, Taoxiang Chen, Biwen Peng, Wanhong Liu, Jian Xu, Yuanteng Fan, Jun Yin, Zhi Zhang, Song Han, XiaoHua He

**Affiliations:** 1 Department of Pathophysiology, Taikang Medical School, School of Basic Medical Sciences, Wuhan University, Wuhan, China; 2 School of Basic Medical Sciences-Weifang Joint Research Center for Pediatric Neurological Diseases and Innovation and Transformation, Wuhan University, Wuhan, China; 3 Hubei Provincial Key Laboratory of Developmentally Originated Disease, Taikang Medical School, School of Basic Medical Sciences, Wuhan University, Wuhan, China; 4 Department of Research and Development, Wuhan Institute of Biological Products, Wuhan, China; 5 Department of Research and Development, Yujin Bio-Pharma Wuhan CNBG Co., Ltd, Wuhan, China; 6 Department of Physiology, Taikang Medical School, School of Basic Medical Sciences, Wuhan University, Wuhan, China; 7 Department of Immunology, Taikang Medical School, School of Basic Medical Sciences, Wuhan University, Wuhan, China; 8 WeiFang Maternal and Child Health Hospital, Weifang, China; 9 Institute of Pediatrics, Weifang Medical University, Weifang, China; 10 Department of Neurology, Zhongnan Hospital, Wuhan University, Wuhan, China

**Keywords:** Multiple sclerosis, Experimental autoimmune encephalomyelitis, Th1 cells, Th17 cells, Anti-human T lymphocyte porcine immunoglobulin, Immunology, Neuroscience, Neurological disorders, Autoimmune diseases

## Abstract

Anti-human T lymphocyte porcine immunoglobulin (P-ATG) is a type of anti-thymocyte globulin (ATG) drug that has been used as an immunosuppressant for severe aplastic anemia and graft-versus-host disease for decades. However, the potential application of ATG for the direct treatment of Multiple sclerosis (MS) and the immune regulatory mechanisms are still unclear. In this study, the therapeutic effects and possible underlying mechanisms of P-ATG were evaluated in the rat model of experimental autoimmune encephalomyelitis (EAE), the animal model of MS. P-ATG could bind to T cells in both human and rat PBMCs in vitro and inhibit inflammatory cytokines secretion from stimulated T cells. P-ATG treatment effectively ameliorated behavioral and pathological alterations in EAE rats through reducing inflammatory infiltration and demyelination in the spinal cords. Moreover, P-ATG treatment significantly suppressed the immune response of T cells, as indicated by a decrease in the IFN-γ^+^ Th1 cell and IL-17^+^ Th17 cell proportions. These findings demonstrated that P-ATG attenuated EAE in rats by inhibiting the activation of helper T lymphocytes from naive T cells to Th1 and Th17 subsets. The therapeutic effect of P-ATG on EAE was independent of T lymphocyte depletion.

**Supplementary Information:**

The online version contains supplementary material available at 10.1038/s41598-026-55508-7.

## Introduction

Multiple sclerosis (MS) is a type of autoimmune disease characterized by recurrent and progressive demyelination, loss of oligodendrocytes, axonal pathology and infiltration of myelin-specific pathogenic T cells into the central nervous system (CNS), leading to demyelination and neuronal injury^1–3^. The pathogenesis of MS has not been fully defined, but some studies suggest that T lymphocytes play an important role in the autoimmune response to MS^4,5^. Previous studies have demonstrated that pathogenic Th1 and Th17 cells are crucial for the pathogenesis of both MS and experimental autoimmune encephalomyelitis (EAE). As the most commonly used animal model of MS, EAE recapitulates many of disease features in humans, such as the invasion of inflammatory T cells in CNS^6–8^. Antigen-specific Th1 and Th17 cells in the CNS secrete large amounts of proinflammatory cytokines, such as interferon-γ (IFN-γ) and interleukin-17 (IL-17), during the progression of EAE and MS ^9,10^. IFN-γ can activate dendritic cells (DCs), monocytes, macrophages, and microglia, while IL-17 can stimulate chemokine production to recruit neutrophils into lesions to enhance the immune response in the CNS^11^.

Anti-thymocyte globulin (ATG) drugs, which are polyclonal antibodies that can induce broad T lymphocyte depletion^12^, have been used clinically for the treatment of graft versus host disease (GVHD)^13^, acute rejection in organ transplantation^14^, and autoimmune diabetes^15^. Previous studies have suggested that the immunosuppressive effect of these drugs is due to T-cell depletion in blood and peripheral lymphoid tissues through complement-dependent lysis^16^.

Currently, ATG is considered to be an immunomodulatory therapy that can ablate T cells and eliminate immunological memory. Commercial ATG drugs for different species of immunized animals include horse ATG, rabbit ATG and porcine ATG (P-ATG)^17,18^. P-ATG and rabbit ATG thymoglobulin are the only two ATGs used for severe aplastic anemia (SAA) treatment in China^19^. P-ATG was reported to achieve better time-related efficacy compared to thymoglobulin for the treatment of SAA^19^. As the unique porcine ATG has been proven clinically in China, P-ATG is believed to be more analogous to human IgG than to ATG from other species. However, the mechanisms of P-ATG in combination with immunotherapies have been poorly studied.

For MS regimens, followed by hematopoietic stem cell transplantation (HSCT) with a graft depletion of immune cells, ATG treatment was applied before transplantation to reconstruct the immune system that no longer induces CNS-directed autoimmunity but provides protective immunity^20^. However, the direct therapeutic potential of ATG for MS remains undetermined. Additionally, the mechanisms by which ATG modulates immune tolerance in the CNS remain unclear. In this study, we found that P-ATG treatment suppressed the immune response in the CNS of EAE rats, and improved demyelination by inhibiting the activation of helper T lymphocytes from naive T cells to Th1 and Th17 subsets, thereby attenuating the development of EAE.

## Results

### P-ATG binds to human and rat CD3^+^ cells in vitro

**Fig. 1:**
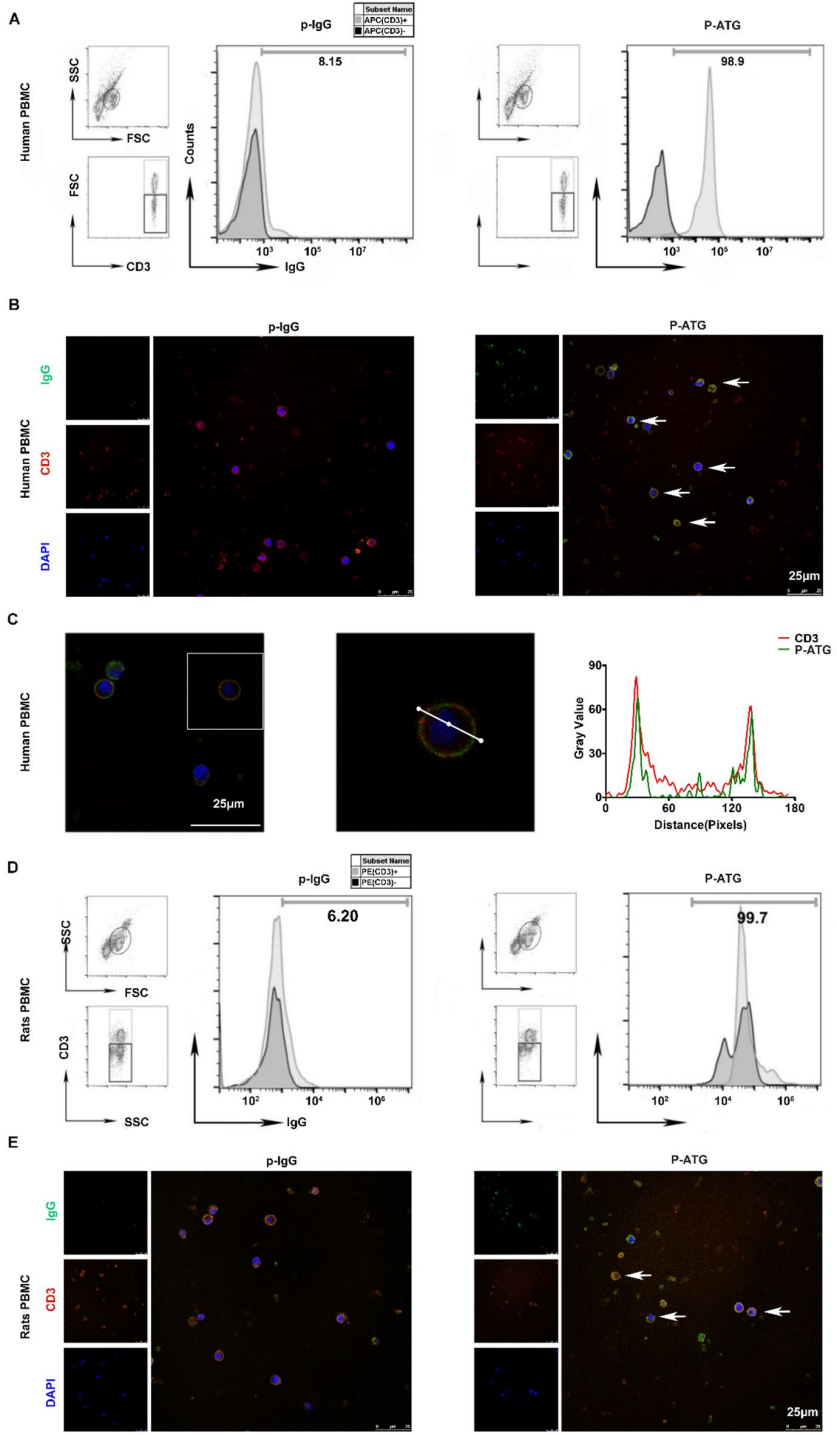
P-ATG binds to CD3^+^ cells in human and rat PBMCs. (**A**) Flow cytometry analysis of porcine IgG^high^ cells in p-IgG and P-ATG treated human PBMCs. After being stained with porcine IgG antibodies, the cells were obtained and subjected to analysis using a flow cytometer. (**B**) Confocal images showing the distribution of porcine IgG (green) binding to cells, human CD3 (red) and DAPI-stained nuclei (blue) in the p-IgG group and P-ATG group (left panel). Confocal images also demonstrated that IgG was mainly localized to the cell membrane, and IgG and CD3 (arrows) were colocalized in PBMCs (right panel). Scale bar = 25 μm. (**C**) Merged images of the indicated CD3^+^ T cells and P-ATG showing complete colocalization (C, left). A side overlap of two peaks was considered partial colocalization (C, right). The image shown was analyzed from five independent experiments. (**D**) Flow cytometry analysis of the number of porcine IgG^high^ cells among 2 groups of rat PBMCs. (**E**) Confocal images showing the distribution of pig-IgG-labeled (green), rat CD3 (orange) and DAPI stained nuclei (blue) in the p-IgG group and P-ATG group (left panel). The images show similar results to those for human PBMCs. Scale bar = 25 μm. All the representative results above are from three independent experiments.

ATG has been reported to contain antibodies that bind to T lymphocyte-specific antigen molecules such as CD3 and CD152^12^. In this study, immunofluorescence labeling was performed to determine whether P-ATG could effectively bind to CD3 in vitro. First, double immunofluorescence labeling of CD3 and porcine IgG was performed on human PBMCs pretreated with P-ATG or p-IgG. The flow cytometry results showed that the fluorescence intensity of IgG in CD3-positive subsets of human PBMCs was elevated in the P-ATG treatment group compared to the p-IgG group (Fig. 1A). Furthermore, the subcellular localization of P-ATG was detected by using laser confocal microscopy. Fluorescence imaging of P-ATG-treated human PBMCs revealed that porcine IgG (green) was located mainly on the cell membrane surface and colocalized with the CD3 protein (red), whereas no fluorescence signal was detected for porcine IgG (green) in p-IgG-treated cells (Fig. 1B). The colocalization of P-ATG and CD3 was assessed by confocal microscopy, and the gray values of CD3 and P-ATG were analyzed^21^(Fig. 1C). Moreover, immunofluorescence labeling of CD3 and porcine IgG was performed on P-ATG- and p-IgG-pretreated rat PBMCs, and the results indicated that only P-ATG was capable of binding to rat CD3^+^ T cells (Fig. 1D-E).

Additionally, the binding affinity of P-ATG to CD3^−^ cells including B cells, NKT cells, and macrophages was also investigated. And the results showed that P-ATG exhibited high binding affinity to B cells, while showing minimal binding to NKT cells or macrophages (Figure S1).

### P-ATG reduces the secretion of inflammatory cytokines

**Fig. 2:**
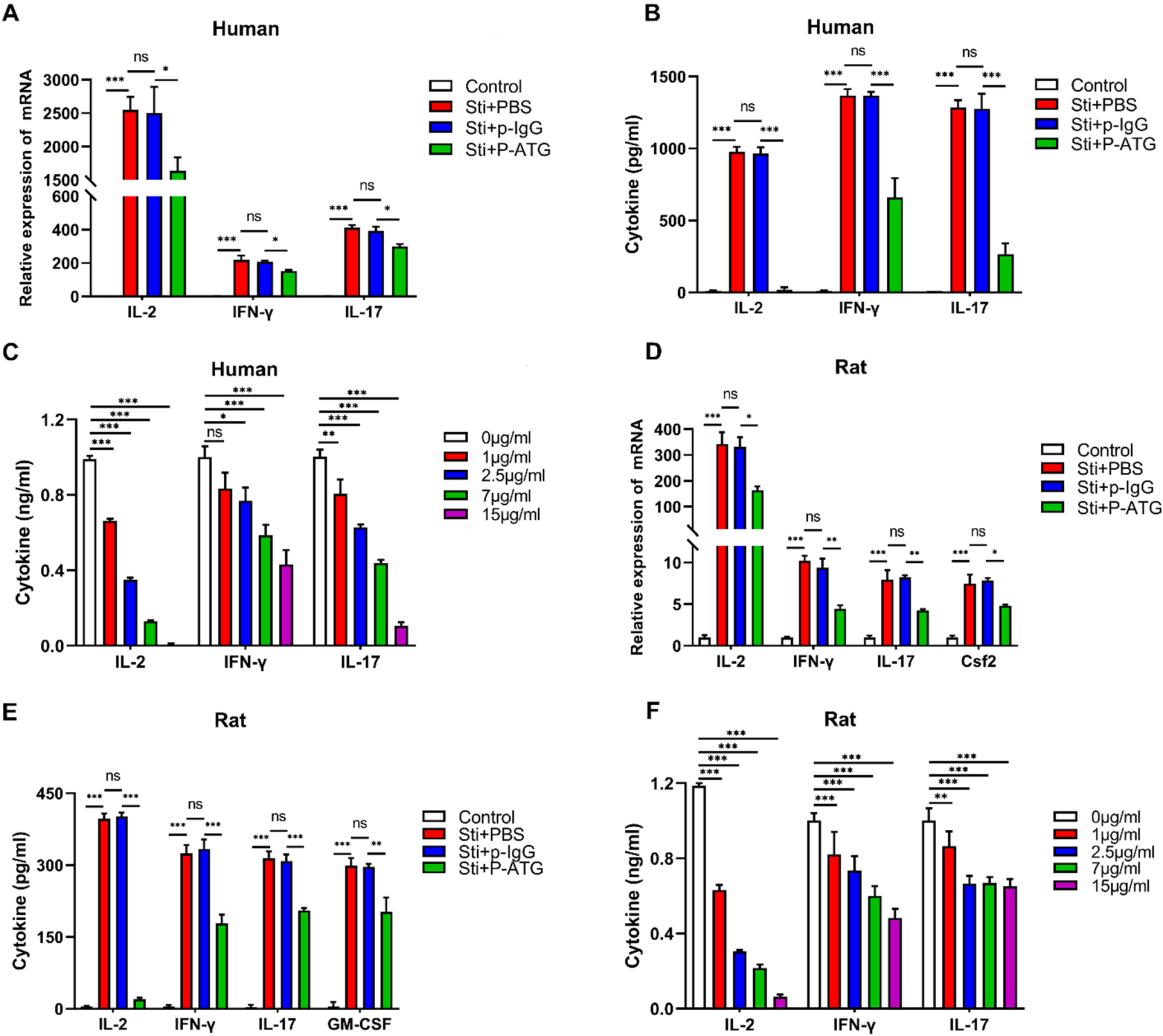
P-ATG inhibited inflammatory cytokine production in a dose-dependent manner in vitro. (**A**) The gene expression of IL-2, IFN-γ, and IL-17 in the cell culture of 4 groups of human PBMCs were measured via qPCR. (**B**) The concentrations of the cytokines IL-2, IFN-γ, and IL-17 in the cell culture supernatants of 4 groups of human PBMCs were measured via ELISA. (**C**) The concentrations of the cytokines IL-2, IFN-γ and IL-17 in the cell culture supernatant from 5 different doses of P-ATG-pretreated human PBMCs were measured via ELISA. The data are presented as the means ± SEMs from two independent experiments with 3 volunteers per group (*n* = 3). (**D**) The gene expression of IL-2, IFN-γ, and IL-17 in the cell culture of 4 groups of rat PBMCs were measured via qPCR. (**E**) The concentrations of the cytokines IL-2, IFN-γ, IL-17 and GM-CSF in the cell culture supernatant were measured by ELISA in 4 groups of rat PBMCs. (**F**) The concentrations of the cytokines IL-2, IFN-γ and IL-17 in the cell culture supernatant from 5 different doses of P-ATG-pretreated rat PBMCs were measured via ELISA. The data are presented as the means ± SEMs from two independent experiments with 4 rats per group (*n* = 4). *P < 0.05, **P < 0.01, ***P < 0.001.

To determine the immunosuppressive effects of P-ATG on inflammatory cytokines, qPCR and ELISAs were performed to evaluate the mRNA levels and the concentrations of cytokines secreted by human PBMCs stimulated with PMA and ionomycin. The results showed that both the mRNA expression and protein levels of the cytokines IL-2, IFN-γ and IL-17 were increased after stimulation with PBMCs for 24 h (*P* < 0.05), and P-ATG pretreatment reduced the levels of these cytokines in a dose-dependent manner (Fig. 2A-C). In addition, P-ATG pretreatment also reduced the mRNA and protein levels of IL-2, IFN-γ, and IL-17 in the culture medium of rat PBMCs stimulated with PMA and ionomycin (Fig. 2D-F). These results demonstrated that P-ATG could inhibit the secretion of inflammatory cytokines such as IL-2, IFN-γ and IL-17 in both human and rat T lymphocytes after inflammatory stimulation. Furthermore, we investigated the inhibitory effect of P-ATG on Th2 cells following PMA and ionomycin stimulation in vitro. The results demonstrated that P-ATG can also inhibit the secretion of Th2-associated cytokines including IL-4, IL-5, and IL-13 (Figure S2).

### P-ATG alleviates inflammatory injuries in EAE rats

**Fig. 3:**
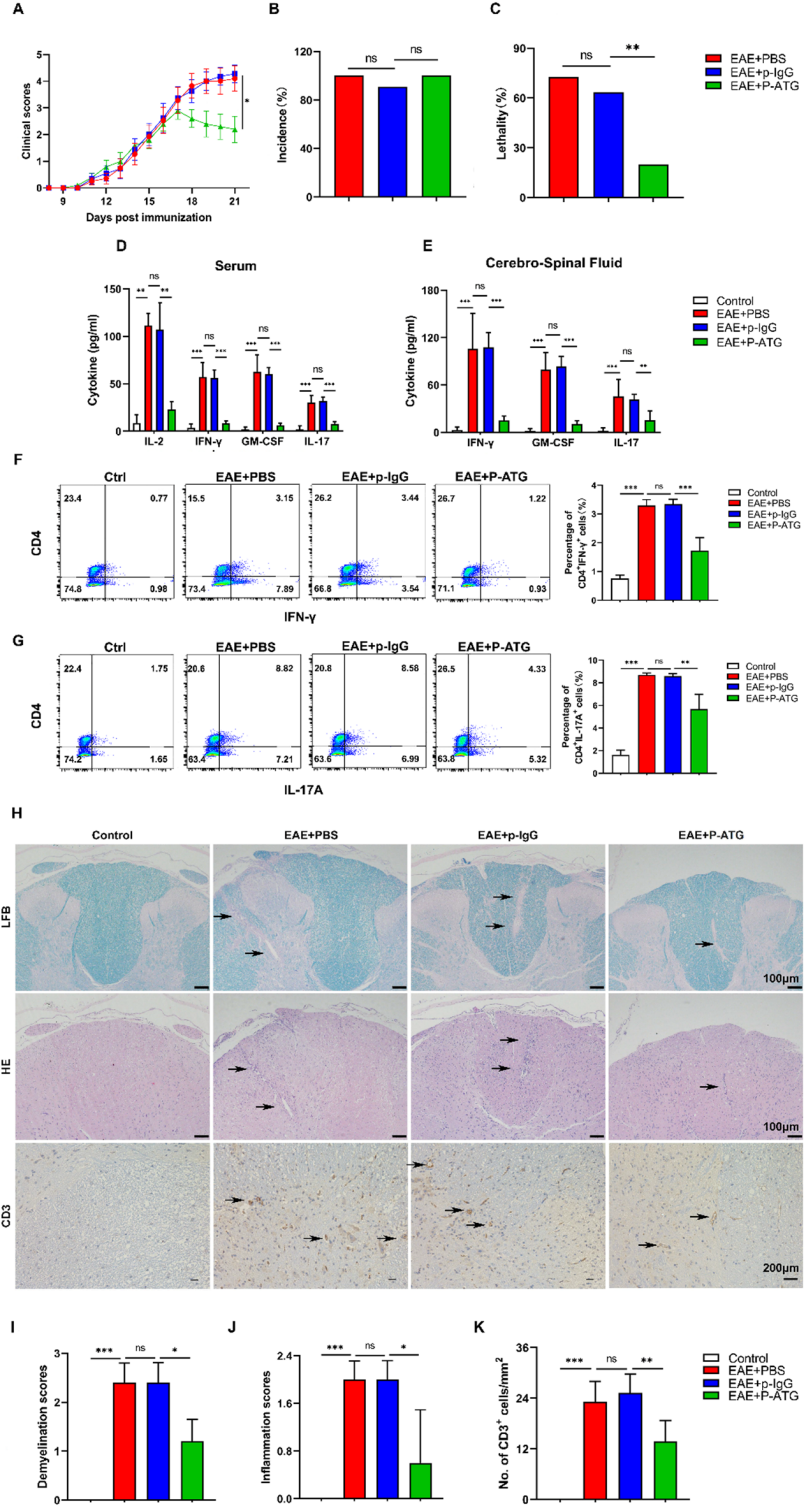
P-ATG treatment alleviates the severity of EAE in rats. (**A**) The mean clinical scores of each group of rats (*n* = 14 each group, *P < 0.05). (**B**) Incidence of disease severity at the end of the experiments. Disease severity was graded as severe (clinical score > 3), moderate (clinical score 1.5–3), or mild (clinical score < 1.5). (**C**) The mortality of rats in each group. (**D**) The concentrations of IL-2, IFN-γ, GM-CSF and IL-17 in the serum were measured via ELISA. (**E**) The concentrations of IFN-γ, GM-CSF and IL-17 in CSF samples were measured via ELISA. (**F-G**) The proportions of Th17 and Th1 cells in each group were detected by flow cytometry, and their proportions were calculated. PBMCs from each group of rats were tested through flow cytometry (*n* = 3). (**H**) LFB (top panel) analysis of spinal cord sections showing the areas of intact myelin (blue) and demyelination (white). The arrows indicate demyelination in the spinal cord. Bar = 100 μm. HE (middle panel) staining of the spinal cord sections showing the infiltration of inflammatory cells in the white matter. The inflammatory cells are shown with arrows. Scale bar = 100 μm. Sections from rats in each group were subjected to immunohistochemistry (bottom panel) using an anti-CD3 antibody, and stereological counts of CD3^+^ cells (arrows) in each group are shown. The CD3^+^ cells are shown with arrows. Scale bar = 100 μm. (**I-J**) The mean pathological scores for inflammation and demyelination in each group were measured by semiquantitative analysis. (**K**) The graph shows the mean number of CD3^+^ cells per square millimeter. The data are expressed as the mean ± SEM; one-way ANOVA of four to five rats per group was used. *P < 0.05, **P < 0.01, ***P < 0.001.

After investigating the immunosuppressive effects of P-ATG on T-cell activation in vitro, we evaluated the potential therapeutic effects of P-ATG on the development and progression of EAE in rats. The results showed that the clinical symptoms of EAE rats began to develop on the 11th day after the first immunization, and the clinical scores of the EAE rats treated with P-ATG were significantly lower than those of the p-IgG group (*P* < 0.05) from the 19th to 21st day after the first immunization; moreover, the clinical scores did not significantly differ between the p-IgG group and the PBS group (Fig. 3A). In addition, the incidence of EAE did not change after P-ATG treatment (Fig. 3B), while P-ATG treatment decreased the lethality rate compared with that in the p-IgG group (from 63.63% to 21.43%) (Fig. 3C). In addition, the efficacy of P-ATG treatment in EAE rats during the acute phase was also evaluated, and the results showed that the EAE rats recovered at about day 41 after immunization, whereas P-ATG treatment group almost fully recovered at day 35 (Figure S3A). The statistical analysis indicated that P-ATG treatment significantly reduced the duration course of the acute phase (Figure S3B).

Furthermore, the concentrations of inflammatory cytokines in the peripheral blood and cerebrospinal fluid of rats in each group were evaluated. The results showed that the levels of IL-2, IFN-γ, GM-CSF and IL-17 in the peripheral blood of the EAE group were greater than those in the control group (*P* < 0.05) (Fig. 3D). The concentrations of these cytokines in the peripheral blood did not show a significant difference between the p-IgG group and the PBS group. However, the levels of cytokines were significantly lower in the P-ATG group compared those in the p-IgG group (Fig. 3D). Similarly, P-ATG treatment also reduced the levels of IFN-γ, GM-CSF and IL-17 in the cerebrospinal fluid of EAE rats (Fig. 3E). To investigate whether P-ATG can inhibit the T cell mediated inflammation in EAE, we checked the alterations of pathogenic Th1 and Th17 cell proportion in rat PBMCs after P-ATG treatment through flow cytometry. The data showed that P-ATG treatment significantly reduced the number of IFN-γ^+^ and IL-17 A^+^ Th cells in the PBMCs of EAE rats (Fig. 3F-G).

At the peak stage of EAE (day 21), spinal cord tissues were dissected and stained with hematoxylin–eosin (HE) or luxol fast blue (LFB) to determine the effects of P-ATG treatment on the severity of inflammation and demyelination. LFB staining demonstrated that demyelination in the white matter of the spinal cord was significantly reduced in the P-ATG-treated group compared to the p-IgG group (Fig. 3H). The mean demyelination score of the EAE + PBS group was 2.4 ± 0.80, the mean demyelination score of the EAE + p-IgG group was 2.4 ± 0.56, and the mean demyelination score of the P-ATG-treated group was 1.2 ± 0.40 (Fig. 3I). HE (Fig. 3J) and CD3 (Fig. 3K) staining also showed that inflammation in the spinal cords of EAE rats was reduced after P-ATG treatment. To further evaluate the effect of P-ATG treatment on anti-CNS immune responses, we examined the infiltration of CD4^+^ and CD8^+^ T cells into the CNS (Figure S4). And the results showed that the number of CD4^+^ and CD8^+^ T cells significantly increased in the spinal cords of EAE rats, whereas the P-ATG treatment reduced the infiltration of these T cells into the spinal cords (Figure S4). These results indicate that P-ATG treatment alleviates the severity of EAE by inhibiting T-cell-associated inflammatory responses in the CNS.

Furthermore, we evaluated the axonal damage in EAE rats through APP immunohistochemical staining, and found that P-ATG treatment significantly attenuated axonal damage in the spinal cords of EAE rats (Figure S5).

### P-ATG has no effect on T-cell activation markers or apoptosis in human PBMCs

**Fig. 4:**
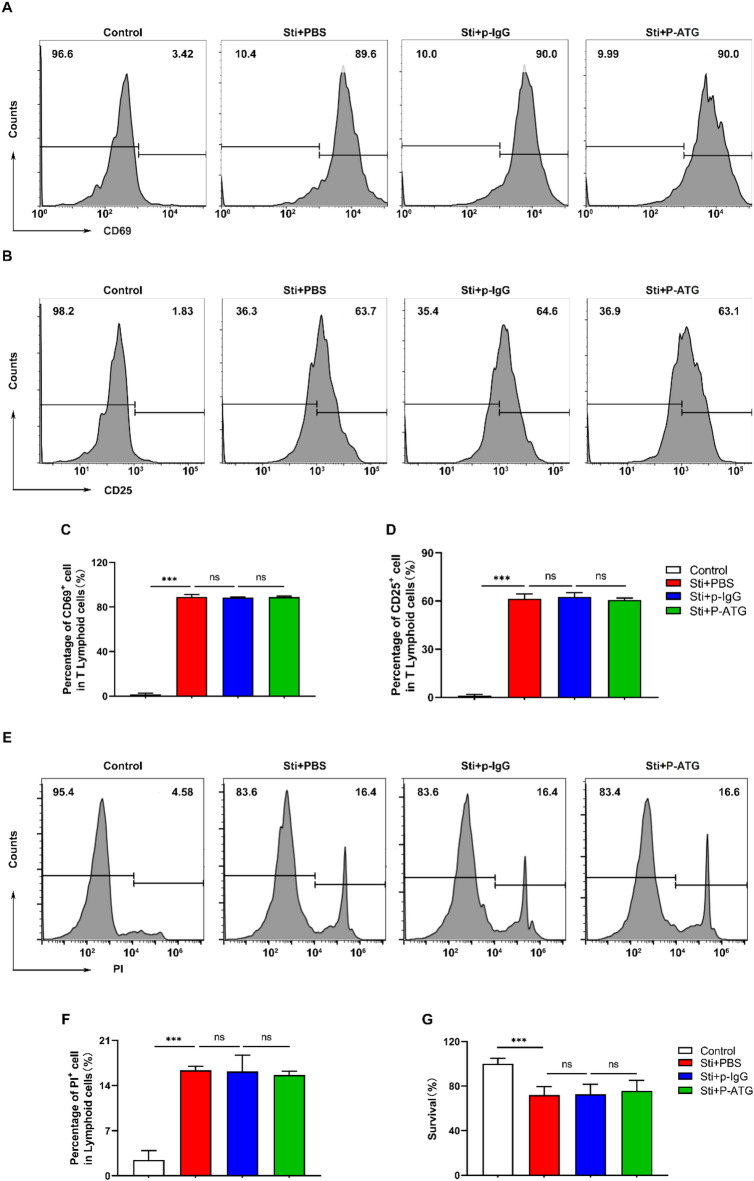
The effects of P-ATG on T cell activation and apoptosis in vitro. (**A**) Flow cytometry analysis of CD69 expression on human CD3^+^ cells (% positive cells). (**B**) Flow cytometry analysis of CD25 expression on human CD3^+^ cells (% positive cells). (**C-D**) Quantitative analysis of the percentage of CD69- and CD25-positive human CD3^+^ cells. (**E**) Flow cytometry analysis of PI expression in human CD3^+^ cells (% positive cells). (**F**) Quantitative analysis of the percentage of human CD3^+^ cells positive for PI. (**G**) Cytotoxicity of P-ATG in human PBMCs was quantified with a CCK-8 assay, while PMA (50 ng/ml) and ionomycin (1 µg/ml) were added for 24 h. Data are presented as the mean ± SEM from three independent experiments. ***P < 0.001.

To further investigate the immunosuppressive effects of P-ATG, human PBMCs were isolated to evaluate the effects of P-ATG on inflammatory T-cell activation and apoptosis. First, multiple immunofluorescence labeling of CD3 and the T-cell activation markers CD69 and CD25 were used to test whether P-ATG had any effect on T lymphocyte activation^22,23^. The flow cytometry results showed that the expression of CD69 and CD25 on CD3^+^ T lymphocytes was significantly increased after 24 h of costimulation with PMA and ionomycin (Fig. 4A-B). However, there was no difference in the expression of CD69 or CD25 between the P-ATG-treated group and the other stimulation groups (Fig. 4C-D). Furthermore, CD3^+^ T lymphocyte apoptosis was detected by double immunofluorescence staining for PI and CD3. The results showed that the number of PI^high^ cells was higher in the stimulation group compared to the control group (Fig. 4E), whereas no significant difference was observed between the P-ATG-treated group and the p-IgG group (Fig. 4F). In addition, the CCK-8 results showed that P-ATG treatment did not affect the survival of PBMCs after stimulation, which was consistent with the results of PI labeling (Fig. 4G). These results indicate that P-ATG has no effect on the T cell activation markers CD69 and CD25 and cannot induce apoptosis in T cells at the same dose that inhibits inflammatory cytokine secretion.

### P-ATG reduces IFN-γ^+^ and IL-17^+^ cells in human PBMCs in vitro

**Fig. 5:**
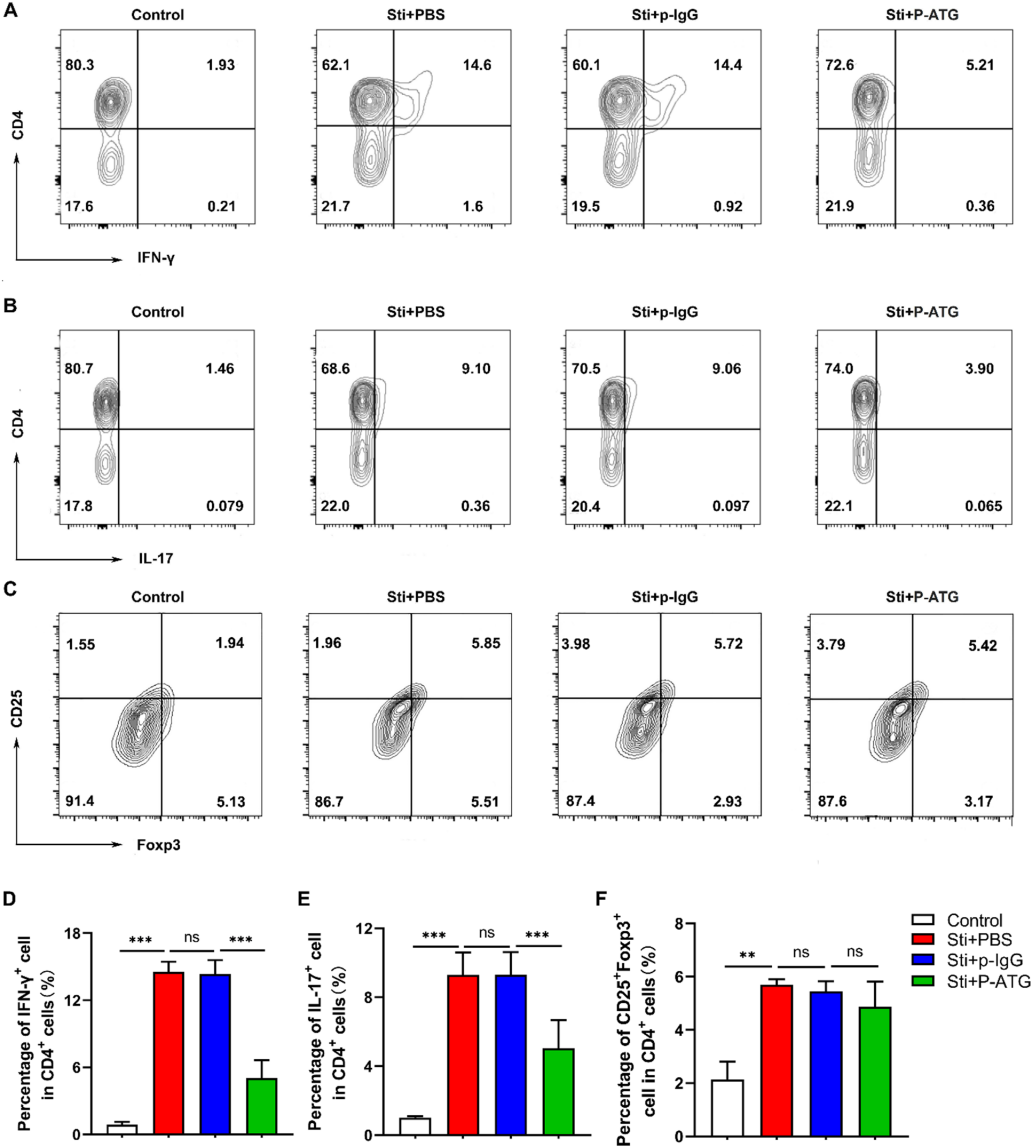
P-ATG reduces the population of Th1 and Th17 cells among stimulated human CD4^+^ T cells (**A-C**) Human PBMCs were stimulated with PMA (50 ng/ml) and ionomycin (1 µg/ml) for 24 h. Subsets of Th1 (IFN-γ^+^CD4^+^) and Th17 (IL-17^+^CD4^+^) cells were analyzed via flow cytometry after intracellular staining for IFN-γ and IL-17. Subsets of Treg cells were analyzed after staining CD25 and Foxp3. (**D-F**) Histograms showing the percentages of IL-17^+^ cells, IFN-γ^+^ cells, and CD25^+^ Foxp3^+^ cells among 1 × 10^5^ CD4^+^ cells. IL-17^+^CD4^+^ cells are Th17 cells, and IFN-γ^+^CD4^+^ cells are Th1 cells, and CD25^+^ Foxp3^+^ cells are Treg cells. The data are representative of three independent experiments (mean ± SEM). *P < 0.05, **P < 0.01, ***P < 0.001.

Since IFN-γ and IL-17 promote inflammation in EAE, we examined the inhibitory effect of P-ATG on IFN-γ^+^ and IL-17^+^ cells in stimulated human PBMCs. The results showed that PMA and ionomycin stimulation increased the expression levels of IFN-γ and IL-17 on CD4^+^ lymphocytes (Fig. 5A-B). There was no difference in the ratio of CD4^+^ lymphocytes expressing high levels of IFN-γ or IL-17 between the p-IgG group and the PBS group. However, P-ATG treatment led to a decrease in the levels of IFN-γ and IL-17 in CD4^+^ lymphocytes (Fig. 5D-E). In addition, the effect of P-ATG on Treg cells in stimulated human PBMCs was also investigated, and we found that P-ATG treatment did not alter the proportion of CD25^+^ Foxp3^+^ Treg cells after stimulation (Fig. 5C, F). Furthermore, the T cell population after stimulation was not reduced following P-ATG treatment in comparison with p-IgG treatment (Fig. S6), suggesting that the reduction in cytokine levels with P-ATG treatment was not due to the alteration of T cell numbers.

### P-ATG has an immunosuppressive effect on rat PBMCs in vitro

**Fig. 6:**
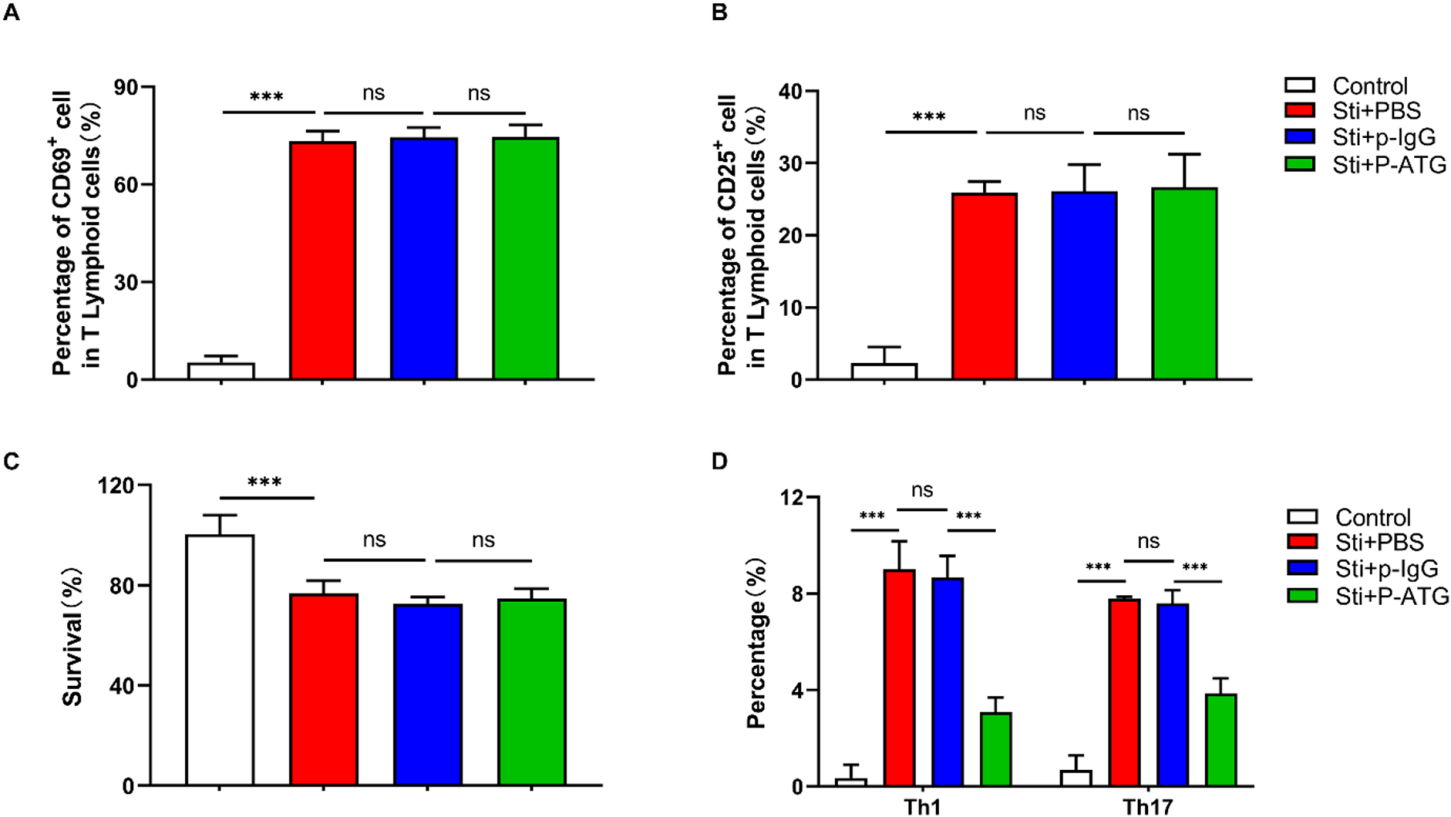
The immunosuppressive effects of P-ATG on stimulated rat T cells in vitro. (**A**) Flow cytometry analysis of CD69 expression on rat CD3^+^ cells (% positive cells). (**B**) Flow cytometry analysis of CD25 expression on rat CD3^+^ cells (% positive cells). (**C**) Histograms showing the percentages of IL-17^+^ cells and IFN-γ^+^ cells among 1 × 10^5^ CD4^+^ cells. (**D**) Cytotoxicity of P-ATG in rat PBMCs was quantified with a CCK-8 assay after treatment with PMA (50 ng/ml) and ionomycin (1 µg/ml) for 24 h. The data are presented as the mean ± SEM from three independent experiments. ***P < 0.001.

To verify the immunosuppressive effects of P-ATG on T lymphocytes, we performed similar experiments to assess T-cell activation and activation, as well as cytotoxicity in rat PBMCs. Consistent with the effects of P-ATG on human PBMCs, P-ATG had no effect on the T cell activation markers CD69 and CD25 after rat PBMC activation (Fig. 6A-B), and was also not toxic to these cells in vitro (Fig. 6C). Moreover, P-ATG significantly inhibited Th1 and Th17 cell activation after rat PBMC activation (Fig. 6D).

## Discussion

To date, the pathogenesis of MS has not been fully defined, and it may be related to immune, genetic and environmental factors^24,25^. Currently, the treatments for MS fall into two broad categories: palliative care and symptomatic treatment. Among them, the main treatments are immune-modulatory drugs, including the first-line drugs IFN-β, natalizumab, Fingolimod and glatiramer acetate. The effects of these drugs include preventing lymphocyte adhesion to the blood‒brain barrier^26^, inhibiting the production of cytokines^27^, preventing lymphocyte migration and inhibiting lymphocyte infiltration of Th1/Th17 cells and B cells into the CNS^28,29^. Although these drugs have achieved short-term effects in the treatment of MS, the long-term use of these drugs reducing the efficacy and enhancing the side effects is still a problem that cannot be ignored^30,31^.

EAE is a model of neurological autoimmune disease mainly mediated by T lymphocytes. It is produced by immunizing susceptible animals with neuronal antigens. The clinical manifestations, immunity and pathology of EAE are similar to those of other neurological autoimmune diseases, such as multiple sclerosis and neuromyelitis optical^32^. At present, the EAE is a recognized ideal animal model for multiple sclerosis and is highly valuable for elucidating the pathogenesis of diseases and evaluating the therapeutic effects of drugs^7^.

ATG is a polyclonal antibody preparation that functions as an immunosuppressive agent. It is produced by immunizing healthy animals (such as horses, rabbits, and pigs) with human thymus or lymphocytes. After immunization, the immunoglobulin was separated and purified from the antiserum of the immunized animals^33,34^. Since the 1960s, these drugs have been used for the first time in the treatment of organ transplant rejection. Currently, ATG is mainly used to treat SAA and GVHD.

With the development of MS research and stem cell transplantation technology, HSCT has emerged as a promising therapeutic option for refractory MS. Since the 1990s, clinical trials have documented the use of ATG combined with cyclosporine A (CsA) as a pretreatment for HSCT for multiple sclerosis. While the pharmacological function of ATG in this therapy is to mitigate host immune rejection and enhance the success rate of stem cell transplantation, its direct therapeutic effect on MS pathology remains unestablished. The efficacy of ATG is clear and safe; however, there is a lack of published reports the long-term efficacy of this drug.

Previous studies have demonstrated that the immunosuppressive effects of ATG on the immune system include the following: (1) T-cell depletion in blood and peripheral lymphoid tissues through complement-dependent lysis and T-cell activation and apoptosis; (2) modulation of key cell surface molecules that mediate leukocyte/endothelial interactions; (3) induction of apoptosis in B-cell lineages and myeloma cell lines; (4) interference with DC functional properties; and (5) induction of Treg and NK-T cells^34^. However, whether ATG can show direct therapeutic effects on EAE and the related mechanism remains still unclear.

In this study, we investigated the effect of P-ATG on EAE rats, and demonstrated that P-ATG can alleviate behavioral abnormalities, reduce behavioral scores, and inhibit inflammatory demyelination in the spinal cord in EAE rats. In this work, the subcutaneous route of administration was selected because it allows for a more gradual release and absorption of the drug, thereby maintaining a more stable drug concentration in serum. In contrast, intravenous injection could lead to a sharp rise in drug concentration, thereby engendering a substantial risk of acute toxicity. In addition, other antibody therapeutics have demonstrated high bioavailability following subcutaneous administration^35,36^, suggesting that subcutaneous injection may achieve comparable efficacy to intravenous infusion in multi-dose chronic treatment regimens. Intriguingly, the therapeutic effect of P-ATG on EAE might be independent of T cell depletion, since P-ATG showed no cytotoxicity to PBMCs after stimulation whereas it inhibited Th1 and Th17 cell activation (Figs. 4F-G and 6C). Moreover, in contrast to other immunosuppressive agents that inhibit T cell activation^37,38^, P-ATG had no effect on T cell activation markers CD69 and CD25. These results indicated that P-ATG might inhibit Th1 and Th17 cell activation via novel mechanisms.

In the present study, the observed lack of T cell apoptosis in vitro following P-ATG treatment is likely due to the absence of complement in the culture system. Additionally, the concentrations of P-ATG used in our study were insufficient to induce T cell apoptosis. However, to better illustrate the mechanism of P-ATG in the context of EAE therapy, we should investigate the T cell apoptosis after P-ATG treatment in vivo in future studies. Mechanistically, P-ATG may modulate immune responses in vivo through Fcγ receptor engagement, perturbation of T cell receptor signaling, or regulation of co-stimulatory molecules on antigen-presenting cells, ultimately inhibiting Th1/Th17 differentiation without inducing cell death. Further investigation is required to reveal the precise mechanism, including the effective components of P-ATG and related transcription factors.

Th1 and Th17 cells are considered to play important roles in the pathogenesis of MS^39,40^. IFN-γ is a pro-inflammatory cytokine, and it can activate microglia to become phagocytic and present antigens, and can also enhance the antigen-presenting ability of myeloid cells to restimulate myelin-antigen-specific CD4^+^ or CD8^+^ T cells in the pathological process of MS^41^. IFN-γ enhances the expression of MHC molecules in the CNS and modulates inflammatory cytokines such as IL-2^42^. Studies have shown that the expression of IFN-γ correlates positively with disease severity in MS patients. Th17 cells can secrete proinflammatory cytokines such as IL-17, IL-22 and GM-CSF^43^. As a marker cytokine secreted by Th17 cells, IL-17 is not only present in the blood and cerebrospinal fluid of MS patients, but is also highly expressed in the injured area of the CNS^44^. Relevant reports indicate that IL-17 can promote blood-brain barrier disruption in the pathogenesis of EAE and MS^45^. In this study, we found that the levels of IFN-γ and IL-17 in the peripheral blood and cerebrospinal fluid of EAE rats were elevated compared to the control group. However, there was no significant difference in the expression of related cytokines between the EAE group and the p-IgG group. The levels of these cytokines in the P-ATG group were significantly lower than those in the p-IgG group. In addition, P-ATG also significantly inhibited the activation of activated T lymphocytes into Th1 and Th17 cells in PBMCs in vitro, and reduced the secretion of related inflammatory cytokines.

It is known that Th17 and Treg cells maintain an immune balance between inflammation and immune tolerance. In this work, we also investigated the effect of P-ATG on Treg cells, and found that P-ATG treatment didn’t change the proportion of Treg cells after pro-inflammatory stimulation (Figs. 4F-G and 6C). However, rabbit ATG was reported to increase the number of Treg cells^34^. These data suggested that certain components of porcine ATG might be different from those of rabbit ATG, leading to disparate effects on Treg cells.

This study has several limitations. Firstly, the EAE animal model fails to fully replicate the complex clinical and pathological heterogeneity of human MS. Secondly, the precise mechanisms by which P-ATG modulates immune cell functions remain incompletely elucidated, particularly with regard to its effects on antigen-presenting cells and its key active components. Furthermore, long-term administration may induce anti-drug antibodies, which could compromise both efficacy and safety. Therefore, further validation is required for the clinical translation of P-ATG. Future studies should systematically evaluate the immunogenicity and long-term safety of P-ATG in more clinically relevant models. Moreover, the exploration of strategies, such as combination therapies or humanisation, is advised in order to advance its clinical development.

## Conclusions

Our data demonstrated that P-ATG regulates the inflammatory response by inhibiting the activation of Th1 and Th17 lymphocytes in EAE. These results revealed that ATG plays an essential role in immunosuppression independent of T cell depletion in neuroinflammatory disorders.

## Materials and methods

### Anti-human T lymphocyte Porcine Immunoglobulin (P-ATG)

P-ATG and normal porcine IgG (p-IgG) were obtained from Yujin Bio-Pharma Wuhan CNBG Co., Ltd. P-ATG was purified from swine immunized with human thymocytes. p-IgG is a type of control IgG used for allergen skin tests before the application of P-ATG.

### Animals

Female Sprague‒Dawley (SD) rats aged six to eight weeks (weighing 180–220 g) were purchased from Beijing Vital River Laboratory Animal Technology Co., Ltd. Animals were maintained at the Institute of Model Animals, Wuhan University, under pathogen-free conditions. All the experiments were performed in accordance with the National Institutes of Health Guide for the Care and Use of Laboratory Animals (Institute of Laboratory Animal Resources, Commission on Life Sciences, National Research Council; National Academy Press, Washington, D.C., 2010), with approval from the Institutional Animal Care and Use Committee of Wuhan University.

### EAE induction and treatment

The EAE model was induced as previously described^46^. Before beginning the experiment, 40 healthy SD rats of similar weight were selected and randomly divided into 4 groups (*n* = 10 in each group) by the ear tag method. These SD rats were immunized on days 0 and 7 with 200 µL of an emulsion of rat spinal cord homogenate in phosphate-buffered saline (PBS, 50% w/v) mixed with an equal volume of incomplete Freund’s adjuvant (Sigma‒Aldrich, St. Louis, MO) supplemented with 5 mg/mL *Mycobacterium tuberculosis* (H37RA). In addition, pertussis toxin (PTX, List Biological Laboratories) was administered at a dose of 2 µg per rat intraperitoneally (i.p.) immediately after immunization and again 48 h later. Beginning on the day when the first neurological signs appeared, the animals were subcutaneously injected with p-IgG/P-ATG (20 mg/kg) or an equal volume of PBS once daily. The clinical signs of EAE were monitored daily according to the following clinical scoring system as previously described^46^: 0, no overt signs of disease; 1, limp tail or hind limb weakness but not both; 2, limp tail and hind limb weakness; 3, partial hind limb paralysis; 4, complete hind limb paralysis; and 5, moribund state, euthanized. The rats were euthanized using carbon dioxide following the guidelines from the American Veterinary Medical Association (AVMA). Carbon dioxide was introduced into the chamber at a rate of 30% of the chamber volume per minute until the rats lost consciousness. Subsequently, the concentration of carbon dioxide in the chamber was increased to 80% for 10 min.

### Histopathology and immunohistochemistry

The rats were sacrificed on the 21st day after the first immunization. The spinal cords were removed and immersed in 4% paraformaldehyde for 48 h. Tissues were paraffin-embedded, and samples that contained the lumbar spinal cord regions were sectioned. Sections of the spinal cord were deparaffinized and stained with LFB and HE to assess demyelination and inflammatory infiltration. For immunohistochemistry, sections were incubated with CD3 antibodies (1:200; Thermo Scientific, Waltham, MA) overnight at 4 °C. Detection was performed using HRP-conjugated secondary antibody (1:500, Abcam) and DAB chromogen (Vector Labs), followed by hematoxylin counterstaining, with all sections finally dehydrated, cleared in xylene, and mounted with Permount for microscopic examination.

### Histopathology and immunofluorescence

On day 21 postimmunization, the rats were sacrificed (*n* = 5 per group), and their spinal cords were dissected. The spinal cord tissues were fixed in 10% formalin in PBS and paraffin-embedded. The spinal cord sections were stained with HE and LFB to evaluate inflammation and demyelination. The degrees of inflammation (HE scores) were calculated as follows: 0, normal; 1, cellular infiltrates partially around the meninges and blood vessels; 2, 1–10, lymphocyte infiltrates in the parenchyma; 3, 11–100, lymphocyte infiltrates in the spinal cord; and 4, more than 100, lymphocyte infiltrates in the spinal cord^47^. The severity of demyelination (LFB scores) was analyzed as follows: 0, normal; 1, myelin sheath injuries in rare areas; 2, a few areas of demyelination; 3, confluent perivascular or subpial demyelination; 4, massive demyelination involving one half of the spinal cord^47^; and 5, extensive demyelination involving the whole cord^48^.The spinal cord tissue sections from the individual groups of rats were deparaffinized, rehydrated, and subjected to antigen retrieval. The sections were treated with 3% BSA in PBS and incubated with anti-CD3 (1:500; Thermo Scientific, Waltham, MA) for 12 h at 37 °C. After being washed, the sections were incubated with secondary antibody and costained with DAPI. The signals were examined under a fluorescence microscope (Olympus 1000, Japan).

### Isolation of peripheral blood mononuclear cells (PBMCs)

Blood was collected from control rats and healthy donors, and from EAE rats sacrificed on the 21st day after the first immunization, with the treatment of PBS, p-IgG or P-ATG. Human peripheral blood was donated by healthy volunteers and used solely as a biological material source for in vitro experiments. All procedures involving human samples were carried out in accordance with the Declaration of Helsinki and relevant institutional guidelines and regulations. The study protocol was approved by the Medical Ethics Committee of Zhongnan Hospital of Wuhan University (Approval No. 2022033 K). Written informed consent was obtained from all human donors prior to blood collection. Whole-blood samples were diluted with sterile PBS, enriched with 70% Percoll (GE HealthCare, Uppsala, Sweden) gradients and centrifuged for 30 min at 1000 × g. For stimulation, PBMCs were treated with 500 ng/mL PMA and 10 µg/mL ionomycin (Sigma‒Aldrich, St. Louis, MO). P-ATG and p-IgG were added 1 h before stimulation.

### Immunofluorescence

To assess the location of P-ATG and p-IgG on PBMCs, the cells were fixed in 4% paraformaldehyde. P-ATG (1:500) and p-IgG (1:500) were used as the primary antibodies and were incubated with PBMCs overnight at 4 °C. Subsequently, cell surface staining was performed with the following Abs: PE-conjugated anti-CD3 (mouse anti-rat, BD, Breda, The Netherlands) for the staining of rat T cells, APC-conjugated anti-CD3 (mouse anti-human, BD, Breda, The Netherlands) for the staining of human T cells, and FITC-IgG (rabbit anti-pig, ThermoScientific, Waltham, MA) for the staining of porcine IgG as a secondary antibody. The cells were washed three times with PBS prior to DAPI (ThermoScientific, Waltham, MA) staining. Finally, cellular colocalization of PBMCs was assessed by using a confocal microscope (Leica Microsystems, Wetzlar, Germany).

### Enzyme-linked immunosorbent assay (ELISA)

PBMCs were stimulated with 500 ng/mL PMA and 10 µg/mL ionomycin as described above. Supernatants from the PBMC cultures were collected 24 h after antigen stimulation. Peripheral blood and cerebrospinal fluid (CSF) samples were collected at the peak of the EAE clinical score. The levels of the cytokines IL-2 (DAKAWE, Beijing, China), IL-17 (DAKAWE, Beijing, China), IFN-γ (DAKAWE, Beijing, China) and GM-CSF (Multi Science, Hangzhou, China) in each sample were measured using the Bradford method according to the manufacturer’s instructions.

### Quantitative PCR

Total RNA from human or rat PBMCs was extracted using TRIzol reagent (Invitrogen, Carlsbad, USA). Then, reverse transcription was performed using the HiScript^®^III RT SuperMix for qPCR (Vazyme, Nanjing, China) according to the manufacturer’s protocol. Quantitative PCR (qPCR) was performed with ChamQ Universal SYBR qPCR Master Mix (Vazyme, Nanjing, China). The expression of target genes was normalized to GAPDH. The 2^−ΔΔCt^ relative quantification method was used to calculate the target genes’ expression levels. A list of primer sequences is provided in Table 1.

**Table 1:** Primer sequences.

Gene	Forward primer (5’-3’)	Reverse primer (5’-3’)
hIL-2	GCACTAAGTCTTGCACTTGTCA	AATGCTCCAGTTGTAGCTGTG
hIL-17 A	CGCTGATGGGAACGTGGACTAC	GGTGGACAATCGGGGTGACA
hIFN-γ	TCGGTAACTGACTTGAATGTCCA	TCGCTTCCCTGTTTTAGCTGC
hGAPDH	GGAGCGAGATCCCTCCAAAAT	GGCTGTTGTCATACTTCTCATGG
mIL-2	GTGCTCCTTGTCAACAGCG	GGGGAGTTTCAGGTTCCTGTA
mIL-17 A	TTTAACTCCCTTGGCGCAAAA	CTTTCCCTCCGCATTGACAC
mIFN-γ	ATGAACGCTACACACTGCATC	CCATCCTTTTGCCAGTTCCTC
mCsf2	GGCCTTGGAAGCATGTAGAGG	GGAGAACTCGTTAGAGACGACTT
mGAPDH	AGGTCGGTGTGAACGGATTTG	TGTAGACCATGTAGTTGAGGTCA
hIL-4	CGGCAACTTTGTCCACGGA	TCTGTTACGGTCAACTCGGTG
hIL-5	TCTACTCATCGAACTCTGCTGA	CCCTTGCACAGTTTGACTCTC
hIL-13	CCTCATGGCGCTTTTGTTGAC	TCTGGTTCTGGGTGATGTTGA

### Flow cytometry

PBMCs were collected from 6-well culture plates. The antibodies used for cell surface staining and intracellular staining are shown in Table 2. The cells were analyzed on a FACS Aria III flow cytometer (BD Bioscience, Breda, The Netherlands). The flow cytometric analysis was performed using Flow Jo (version 9.2; Tree Star, Inc.).

**Table 2:** Antibodies and chemicals.

Name	Conjugate	Host	Reactivity		Company	Cat. no.
Anti-CD3	APC	Mouse		Human	Biolegend	300,311
Anti-CD3	PE	Mouse		Human	Biolegend	317,307
Anti-CD69	FITC	Mouse		Human	Biolegend	310,903
Anti-CD25	APC	Mouse		Human	BD	555,434
Anti-CD4	APC-Cy7	Mouse		Human	BD	561,839
Anti-IFN-γ	Alexa Fluor647	Mouse		Human	BD	557,729
Anti-IL-17	Alexa Fluor647	Mouse		Human	BD	560,490
Anti-CD4	FITC	Mouse		Human	Biolegend	317,407
Anti-CD25	PE	Mouse		Human	Biolegend	302,605
Anti-Foxp3	APC	Mouse		Human	Biolegend	320,113
Anti-CD126	PE/Cy7	Mouse		Human	Biolegend	352,809
Anti-CD3	PE	Mouse		Rat	Biolegend	201,411
Anti-CD4	Alexa Fluor710	Mouse		Rat	Biolegend	203,305
Anti-CD25	APC-Cy7	Mouse		Rat	Biolegend	202,103
Anti-IFN-γ	Alexa Fluor647	Mouse		Rat	Biolegend	507,809
Anti-IL-17	APC	Mouse		Rat/Human	Invitrogen	MAN0007965
Anti-pig-IgG	FITC	Rabbit		Pig	Invitrogen	61-9111
Anti-rabbit-IgG	FITC	Mouse		Rabbit	BBI	D110068
Anti-CD69	Unconjugated	Rabbit		Rat	MultiSciences	70-ab32084-050
DAPI	/	/		/	Invitrogen	S36968
PI	/	/		/	BBI	A601112
Anti-CD3	Unconjugated	Mouse		Human	Proteintech	60181-1-1-Ig
Anti-CD56	Unconjugated	Rabbit		Human	Proteintech	83365-6-RR
Anti-CD20	Unconjugated	Mouse		Human	Proteintech	60271-1-Ig
Anti-CD11b	Unconjugated	Rabbit		Human	Proteintech	21851-1-AP
Anti-pig-IgG	FITC	Rabbit		Pig	Immunoway	RS030618

### Cytotoxicity assay

The cytotoxicity of P-ATG was measured with a Cell Counting Kit-8 (CCK-8; Dojindo, Tokyo, Japan) according to the manufacturer’s instructions. CCK-8 reagent was added to each well, and the cells were incubated for another 1–4 h. The absorbance at 450 nm was measured using a scanning microplate reader (BioTek, Winooski, USA).

### Statistics

Data were tested for normality using the Shapiro-Wilk test. Significant differences between groups were analyzed with either an unpaired t test or one-way analysis of variance (ANOVA) where appropriate. Post hoc analysis was performed with the Newman‒Keuls multiple-comparison test. The EAE clinical scores were analyzed using repeated measures ANOVA. Differences were considered to be significant at a critical value of *P* < 0.05. All values are presented as the mean ± standard error of the mean (SEM).

## Supplementary Information

Below is the link to the electronic supplementary material.

Supplementary Material 1

## Data Availability

The data that support the findings of this study are available from the corresponding author upon reasonable request.
