# Supplementary material for: Anti-human T lymphocyte porcine immunoglobulin attenuates experimental autoimmune encephalomyelitis by inhibiting T cell activation
Source: Sci Rep. 2026 May 30;16:24796. doi: 10.1038/s41598-026-55508-7 (PMC13458704; doi:10.1038/s41598-026-55508-7)
Supplement: Supplementary file 1 [file 41598_2026_55508_MOESM1_ESM.docx]

**Supplementary Materials**

**Figure S1.** The binding affinity of P-ATG on B cells, NKT cells, and macrophages in human PBMCs. (A) Confocal images showing the distribution of P-ATG (green) binding to cells, CD20 (red) and DAPI-stained nuclei (blue) in human PBMCs. (B) Confocal images showing the distribution of P-ATG (green) binding to cells, human CD56 (red) and CD3 (blue) in human PBMCs. (C) Confocal images showing the distribution of P-ATG (green) binding to cells, CD11b (red) and DAPI-stained nuclei (blue) in human PBMCs (scale bar= 25 μm).

**Figure S2.** P-ATG inhibited Th2 cytokine production in vitro. The gene expression of IL-4, IFN-γ, and IL-17 in the cell culture from 4 groups of human PBMCs was measured via qPCR. The data are presented as the means ± SEMs from two independent experiments with 3 volunteers per group (n=3). **P* < 0.05, ***P* < 0.01, ****P* < 0.001.

**Figure S3.** P-ATG treatment reduces the duration of acute phase in EAE rats. (A) Mean clinical scores of each group of rats (n=8 each group). (B) The area under curve of clinical scores of each group of rats. (C) The duration of acute phase in EAE rats from each group. The acute phase is defined as the period of peak disease severity, measured from the onset of clinical signs (clinical score ≥ 1) to the commencement of the remission phase. Data were presented as the mean ± SEM. ***P* < 0.01, ****P* < 0.01.

**Figure S4.** P-ATG inhibited the infiltration of immune cells into the spinal cord of EAE rats. (A) CD4^+^ T cell and CD8^+^ T cell infiltration were shown in the spinal cord of rats (scale bar= 50 μm). (B-C) Density of CD4^+^T cells (B) and CD8^+^T cells (C) was analyzed in the lumbar spinal cord white matter (n=3). Data are presented as the mean± SEM. **P* < 0.05, ***P* < 0.01, ****P* < 0.001.

**Figure S5.** P-ATG reduces axonal damage in the spinal cord of EAE rats. (A) Representative immunohistochemical staining of APP expression in each group (scale bar= 200 μm). (B) Quantification of the immunoreactivity in each group (n=3). Data are presented as the mean± SEM. ***P < 0.001.

**Figure S6.** The effects of P-ATG on T cell population in human PBMCs after stimulation. (A) Human PBMCs were isolated and stimulated with PMA (50 ng/ml) and ionomycin (1 μg/ml) for 24 hours. The number of viable CD3^+^ T cells was detected via flow cytometry. (B) Histograms showing the quantification results of the proportion of CD3^+^ T cells (n=3). The data are presented as the mean± SEM.
